# Immune Checkpoint Inhibitors in the Treatment of HCC

**DOI:** 10.3389/fonc.2020.601240

**Published:** 2021-01-07

**Authors:** Clelia Donisi, Marco Puzzoni, Pina Ziranu, Eleonora Lai, Stefano Mariani, Giorgio Saba, Valentino Impera, Marco Dubois, Mara Persano, Marco Migliari, Andrea Pretta, Nicole Liscia, Giorgio Astara, Mario Scartozzi

**Affiliations:** ^1^ Medical Oncology Unit, University Hospital and University of Cagliari, Cagliari, Italy; ^2^ Medical Oncology Unit, Sapienza University of Rome, Rome, Italy

**Keywords:** Hepatocellular carcinoma, immune checkpoint inhibitors, atezolizumab, pembrolizumab, nivolumab

## Abstract

Hepatocellular carcinoma (HCC) is the typical inflammation-induced neoplasia. It often prospers where a chronic liver disease persists, thus leading a strong rationale for immune therapy. Several immune-based treatments, including immune checkpoint inhibitors (ICI), cytokines, adoptive cell transfer, and vaccines, have been tested in the treatment of HCC. In this review, we summarize the role of the ICI in HCC patients in various sets of treatment. As for advanced HCC, the anti-Programmed cell Death protein 1 (PD1) antibodies and the anti-Cytotoxic T-Lymphocyte Antigen *4* (CTLA-4) antibodies have been examined in patients with enthusiastic results in phase I-II-III studies. Overall, this led the Food and Drug Administration (FDA) to approve pembrolizumab, nivolumab, and nivolumab + ipilimumab in the second-line setting. The anti- Programmed Death-Ligand 1 (PDL-1) antibodies have also been evaluated. Thanks to the results obtained from phase III IMbrave study, atezolizumab + bevacizumab is now the standard of care in the first-line advanced setting of HCC. As for localized HCC, the putative immunological effect of locoregional therapies led to evaluate the combination strategy with ICI. This way, chemoembolization, ablation with radiofrequency, and radioembolization combined with ICI are currently under study. Likewise, the study of adjuvant immunotherapy following surgical resection is underway. In addition, the different ICI has been studied in combination with other ICI as well as with multikinase inhibitors and anti-angiogenesis monoclonal antibody. The evidence available suggests that combining systemic therapies and locoregional treatments with ICI may represent an effective strategy in this context.

## Introduction

Hepatocellular Carcinoma (HCC) claims to be 90% of primary liver cancer and represents the second cause of death due to malignancy in males (1). The triggers most likely involved in cancer development are chronic infections by Hepatitis B or C viruses, diabetes, aflatoxin-B1 (AFB1) exposure, obesity, alcohol abuse, nonalcoholic steatohepatitis (NASH), nonalcoholic fatty liver disease (NAFLD), and metabolic syndrome (2–11).

Indeed, chronic inflammation boosts the tumor immunogenicity and induces hepatocellular DNA damage, genetic and epigenetic mutations. Furthermore, chronic inflammation allows to escape the host immune surveillance in cooperation with an immunosuppressive surrounding (2–8).

The impairment of various immune components promotes tumorigenesis. The liver immune milieu consists of an assortment of innate and adaptive immune cells that undergo alterations that promote cancer development and progression. Immune checkpoints are involved in the inhibition of T- or natural killer cell activation as well as in the initiation and preservation of tumor immune tolerance. B and T cells, natural killer cells, dendritic cells, tumor-associated macrophages, monocytes, and myeloid-derived suppressor cells express on their surface immune-checkpoints and their ligands. The most well-known of them are cytotoxic T-lymphocyte protein 4 (CTLA-4), which promotes immunosuppression, and programmed cell death protein 1 (PD-1) that leads to the T-cell exhaustion status, which inhibits T-cell multiplication and release of cytotoxic mediators (2–8).

**Figure 1:**
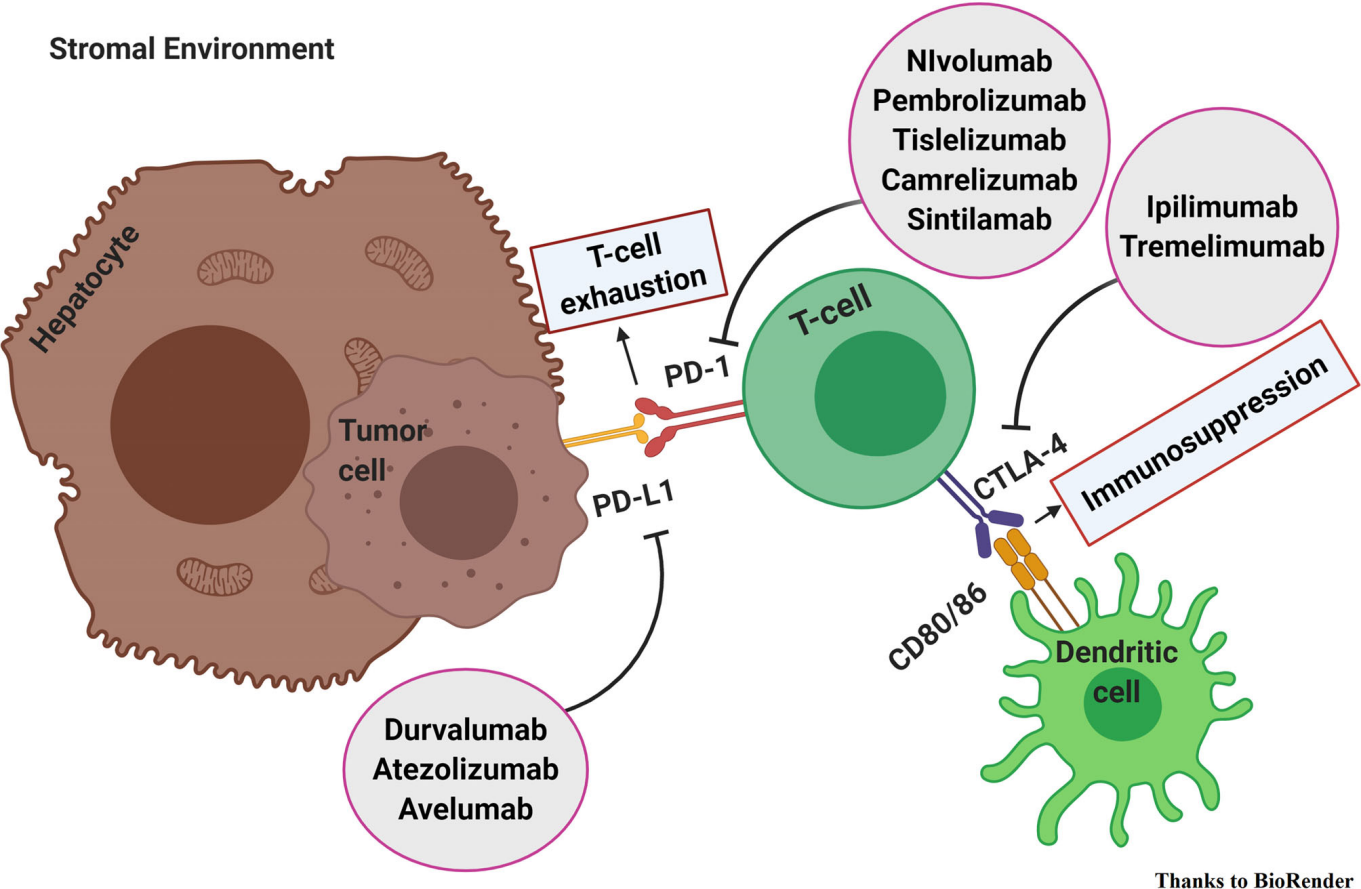
Immune checkpoint inhibitors in hepatocellular carcinoma. PD-1 binding its ligand PD-L1 prevents TCR signaling, blocks T cell proliferation, and induces the exhaustion of T cells. CTLA-4 binds CD80/CD86 and blocks activation of the T cells. The inhibition of immune checkpoints avoids immune exhaustion, reduces Treg activity, and leads to the reactivation of the anticancer immune response.

In a physiological state, antigens are presented to CD4+ T cells that consequently promote the activity of CD8+ T cells. Thus, leading to an upregulation of CTLA-4 and PD-1. Consequently, the immune checkpoints prevent hyperactivation of the immune response. That way, the tolerogenic environment of the liver is preserved. Therefore, HCC is an immunogenic tumor that builds-up in an immune-suppressed microenvironment. In the setting of chronic inflammation, the cancer develops and flourishes thanks to the recruitment of regulatory T cells (Treg), myeloid-derived suppressor cells (MDSCs), and the upregulation of immune checkpoints, CTLA-4 and PD-1. PD-1 binding its ligand PD-L1 prevents TCR signaling, blocks T cell proliferation, and induces the exhaustion of T cells. Tregs constitutively express CTLA-4 and preclude the immune response through it. CTLA-4 binds CD80/CD86, competing with CD28, and blocks activation of the T cells. It appears clear that the inhibition of immune checkpoints avoids immune exhaustion, reduces Treg activity, and leads to the reactivation of the anticancer immune response (2–5). Thus, immune-checkpoint inhibitors (ICI) seem to be promising treatment strategies (**Figure 1**).

## Immune Checkpoint Inhibitors in Advanced HCC

The systemic therapies for patients with HCC in advanced and intermediate stage, according to Barcelona Clinic Liver Cancer (BCLC), refractory to locoregional therapy was limited to sorafenib for a long time (12). Instead, since 2017, several effective systemic therapies have been recommended by the Food and Drug Administration (FDA), thus turning treatment decision making into a challenge. Several TKIs are now available for first-line [sorafenib (13–22), lenvatinib (23, 24)], second and third-line treatment [regorafenib (25), cabozantinib (26)]. Also, a monoclonal antibody [ramucirumab (27)] is available for second-line treatment. In addition, two anti-PD-1 antibodies [nivolumab (28) and pembrolizumab (29)] and the combination anti-PD1 + anti-CTLA4 [nivolumab + ipilimumab (30)] received FDA’s accelerated approval. On the whole, the anti- angiogenesis remains a cardinal point for treatment, whereas the ICI, including anti-PD-1, anti-PDL-1, and anti-CTLA-4, are becoming increasingly important in the therapeutic scenario.

As for anti-CTLA-4, tremelimumab (31) has been evaluated in a phase II, in a non-controlled, open-label, multicenter clinical trial, in patients with HCC not amenable to locoregional treatment and chronic hepatitis C. Tremelimumab showed a good safety profile along with encouraging outcomes in terms of RR (17.6%), disease control rate (DCR) (76,4%) and time to progression (TTP) (6.48 months).

On this basis, tremelimumab, in combination with durvalumab, has been evaluated. A randomized phase II trial (NCT02519348) has been examined tremelimumab and durvalumab as single-agent as well in combination with two different dosage regimens (tremelimumab 300 + durvalumab vs tremelimumab 75 + durvalumab) in advanced HCC patients. A safety profile along with an antitumor activity were demonstrated in the preliminary results, especially for the tremelimumab 300 + durvalumab regimen. Grade 3/4 adverse events were reported in 28.9% of patients (tremelimumab 300 + durvalumab, 35.1%; tremelimumab 75 + durvalumab, 25.6%; durvalumab, 19.8%; tremelimumab, 42%). The ORR observed were the following: 22.7% for tremelimumab 300 + durvalumab; 9.5% for tremelimumab + durvalumab; 9.6% for durvalumab, and 7.2% for tremelimumab (32).

As a result, the data from the phase III Himalaya trial (33) are expected to assess the efficacy of tremelimumab + durvalumab versus sorafenib in the first-line setting of HCC patients not susceptible to locoregional therapy.

As regards anti-PD-1, nivolumab and pembrolizumab have been investigated in phase II (CheckMate 040 and Keynote 224, respectively) and phase III studies (CheckMate 459 and Keynote 240, respectively).

The CheckMate 040 phase I/II non-comparative study evaluated nivolumab in patients with unresectable HCC with or without previous treatment with sorafenib. The phase II study showed a promising ORR of 20% with a median extent response of 9.9 months along with a manageable safety profile. The 9-month overall survival (OS) rate was 74%. On this basis, the FDA speeded up the acceptance of nivolumab for HCC pretreated with sorafenib (29). Conversely, the CheckMate 459 trial phase III study (34) failed to demonstrate improved OS with nivolumab versus sorafenib in this setting. Although the results obtained are impressive, showing improvements in survival and response rate along with a lack of adverse events, they were not statistically significant. Median overall survival was 16.4 months for nivolumab and 14.7 for sorafenib [Hazard Ratio (HR): 0.85 p 0.0752], the ORR was 15% for nivolumab and 7% for sorafenib. Also, nivolumab has been assessed in combination with ipilimumab in the Cohort 4 of Checkmate 040 (30). The ORR was 31% with a median duration of response (DOR) of 17 months; DCR was 49%, and 24 months OS rate was 40%.

Based on these impressive results, the FDA recommended the combination of nivolumab and ipilimumab for HCC patients previously treated with sorafenib.

As regards of pembrolizumab, it has been evaluated in the phase II Keynote 224, non-randomized, multicentre, open-label, in HCC BCLC B-C patients pre-treated with sorafenib. Pembrolizumab demonstrated a manageable safety profile along with antineoplastic activity with an ORR of 17%. On this basis, pembrolizumab received FDA’s accelerated approval, and it has been evaluated versus placebo in pre-treated advanced HCC patients in phase III randomized, placebo-controlled Keynote 240 (35). Pembrolizumab improved OS (13.9 months vs 10.6 months HR: 0.78 p: 0.0238), progression free survival (PFS) (3.0 months vs 2.8 months HR: 0.77 p: 0.022) and ORR (16.9% vs 2.2%) with durable responses (DoR 13.8 months) vs placebo. The study, however, was negative. The outcome measures OS and PFS, although impressive, did not achieve statistical significance. Regarding anti-PDL-1, atezolizumab has been tested as first-line treatment in combination with bevacizumab in the phase Ib GO30140 Study (*NCT02715531*). Patients included in arm A received atezolizumab + bevacizumab IV every three weeks, whereas patients included in arm F were randomized 1:1 and took atezolizumab-bevacizumab (F1) or single-agent atezolizumab (F2). In arm A, the ORR (primary endpoint) was 36%, with 76% of responses still ongoing. In arm F, the primary endpoint was PFS. A statistically significant improvement in median PFS was reached with the combination therapy respect to single-agent atezolizumab (F1: 5.6 versus F2: 3.4 months, HR 0.55, 80% confidence interval (CI), 0.40–0.74, P = 0.0108). As for safety, another one primary endpoint for both arms, any-grade treatment-related adverse events (TRAEs) were 68% in arm F1 and 41% in arm F2 (36).

Another crucial study that represents a turning point in the treatment of HCC was the phase III IMbrave 150 Study. In this randomized, open-label trial, advanced HCC patients were randomized 2:1 to receive atezolizumab + bevacizumab or sorafenib until loss of clinical benefit or unacceptable toxicity. Co-primary endpoints were OS and PFS by independent review facility (IRF)-assessed response evaluation criteria in solid tumors (RECIST) 1.1, whereas key secondary endpoints were IRF-ORR per RECIST 1.1 and IRF-ORR per HCC modified RECIST (mRECIST). The primary data analysis showed the achievement of both co-primary endpoints: in the intent-to-treat (ITT) population, at a median follow-up of 8.6 months, OS HR was 0.58 (95% CI, 0.42, 0.79; P = 0.0006) and PFS HR was 0.59 (95% CI, 0.47, 0.76; P < 0.0001) in the atezolizumab plus bevacizumab arm vs the control arm. ORR was 27% in patients receiving atezolizumab and bevacizumab vs 12% in patients receiving sorafenib (P < 0.0001) per IRF RECIST 1.1 and 33 vs 13% (P < 0.0001) per IRF HCC mRECIST for experimental arm vs control arm, respectively. Median treatment duration was of 7.4 months for atezolizumab, 6.9 for bevacizumab, and 2.8 for sorafenib. Moreover, the association of atezolizumab and bevacizumab was well tolerated and procrastinated time to deterioration (TTD) of the quality of life (QoL) of the patients [median TTD, 11.2 vs 3.6 mo; HR, 0.63 (95% CI: 0.46, 0.85)], physical functioning [median TTD, 13.1 vs 4.9 mo; HR, 0.53 (95% CI: 0.39, 0.73)], and role functioning [median TTD, 9.1 vs 3.6 mo; HR, 0.62 (95% CI: 0.46, 0.84)] compared with sorafenib. Furthermore, the combination therapy postponed TTD in patient-reported symptoms (loss of appetite, fatigue, pain, diarrhea) and led to meaningful clinical symptoms deterioration in a lower proportion of patients. Based on this data, atezolizumab + bevacizumab was approved as the first-line standard of care in advanced HCC (37, 38).

## Immune Checkpoint Inhibitors in Localized HCC

Hepatic resection (HR), liver transplantation (LT), and ablation (39) are treatments with curative intent in HCC, according to the EASL clinical practice guidelines (40).

To date, no therapy has proven to be effective in the adjuvant setting (41, 42). Nonetheless, the promising results of immunotherapy in advanced HCC have led to a growing interest in the adjuvant setting too. It is well-known that the liver has an immune suppressive microenvironment to avoid autoimmune phenomena (43, 44). However, in patients with HCC, persistent inflammatory state upregulates the expression of PD-1 (45) and PD-L1 (46), leading to CD8+ T-cells apoptosis and a decrease of their action against tumor cells (47, 48). Moreover, this effect relates to a poor prognosis and a considerable aggressiveness of the tumor and promotes postoperative recurrences in HCC patients (49–52). An increased PD-1 and PD-L1 expression could provide the rationale for the employment of both PD-1 and PD-L1 ICI as adjuvant treatment in HCC.

Adjuvant Immunotherapy with ICI is currently under investigation in HCC patients who underwent loco-regional treatment and are at high risk of recurrence. Unfortunately, no published randomized trials are yet available.

Nivolumab, an anti-PD-1 monoclonal antibody (mAb), is being assessed in a phase III, multicenter, randomized, double-blind CheckMate 9DX trial (NCT03383458). The study has an estimated enrollment of 530 HCC patients who will randomly receive either nivolumab (arm A) or placebo (arm B) (53).

Pembrolizumab, an anti-PD-1 mAb, is now being studied in a phase III, multicenter, randomized, double-blinded, two-arm study Keynote-937 (NCT03867084). Participants (estimated enrollment: 950 patients) will receive intravenous (IV) pembrolizumab if assigned to arm A, and IV placebo if assigned to arm B.

Durvalumab, an anti-PD-L1 mAb, alone or combined with bevacizumab, is under examination in a phase III, randomized, double-blind, placebo-controlled, multicenter study, EMERALD-2 (NCT03847428), in the same HCC high-risk population of the abovementioned studies. Patients randomized to arm A will receive IV durvalumab plus IV bevacizumab; arm B patients will receive durvalumab plus placebo, and arm C subjects will be assigned two placebos. The estimated enrollment is of 888 participants.

Atezolizumab, an anti-PD-L1 mAb, is under evaluation in association with bevacizumab in phase III, multicenter, randomized, open-label IMbrave050 study (NCT04102098). Patients will be randomly allocated to arm A to receive IV atezolizumab plus IV bevacizumab or to arm B to active surveillance. The study estimates to enroll 662 participants.

Toripalimab, an anti-PD-1 mAb, is under study in a phase II/III, randomized, double-blind, placebo- controlled study, the JUPITER 04 trial (NCT03859128). The estimated 530 participants enrolled will be treated with toripalimab if assigned to arm A, whereas they will not receive it if assigned to arm B.

The primary outcome of these trials is the measure of the recurrence-free survival (RFS), except for Keynote-937, which will consider both RFS and OS. However, it is significant to specify that EMERALD-2 will evaluate only the RFS for arm B versus arm C as primary endpoint, while the RFS for arm A versus arm C represented the secondary endpoint.

Loco-regional treatments in HCC are used in patients with early-stage (0-A BSCL staging) who are not eligible for surgical treatment or transplant, or in patients with advanced-stage (B-C BSCL) not amenable to kinase-inhibitor drugs (Sorafenib or Regorafenib). The most used local procedures are transarterial chemoembolization (TACE) (54–59), radiofrequency ablation (RFA) (60), stereotactic body radiotherapy (SBRT) (61, 62), transarterial radioembolization, and embolization *via* microspheres loaded with yttrium-90 (Y-90) (63–65).

These loco-regional treatments allow to release a high quantity of tumor antigens through the destruction of the tumor cells. For this reason, the effectiveness of their combination with the ICI has been investigated with encouraging results (66).

The results of two studies are currently available. In the study conducted by Duffy et al., 32 patients were started on tremelimumab therapy at two dose levels every four weeks for six administrations total, then followed by 3-monthly infusions until they matched up off-treatment. On the 36th day, subtotal radiofrequency ablation or chemoablation were performed. Of the 19 evaluable patients, 5 (26,3%) reached a firm partial response. Six-week tumor biopsies displayed an increase in CD8+ T cells in patients who presented a clinical benefit alone. For this refractory HCC population, six and twelve- month probabilities of tumor progression-free survival were 57,1 and 33,1%, respectively, with a median time to tumor progression of 7,4 months. The mOS was 12,3 months (67).

Furthermore, the phase II trial by Zao et al. (NCT03939975) assessed the response of 50 HCC patients who progressed to a first-line with sorafenib and started a second-line treatment with anti-PD1 (pembrolizumab or nivolumab). Of these, 33 patients underwent subtotal thermal ablation because the disease did not progress or had an atypical response to anti-PD-1 inhibitor. Additional ablation ameliorated effectiveness with acceptable toxicity, and the RR rose from 10 to 24% (12/50). The median time to progression (MTP), PFS, and OS was 6.1, 5, and 16.9 months, respectively (68).

Currently, there are several trials underway to evaluate which combination is more useful and could allow us to get the best results in terms of ORR.

The combination of ICI with stereotactic radiotherapy (SBRT) is still under study. In particular, the phase II/III trial NCT04167293 (ISBRT01) is evaluating this type of local treatment in association with sintilimab (a monoclonal antibody anti-PD1) in an advanced stage of HCC. Another study is NCT03380130 (NASIR-HCC), a phase II clinical trial that is investigating nivolumab combination in the same patient settings. While phase II study NCT03316872 is studying SBRT combined with pembrolizumab.

The role of TACE in combined therapy is also under study. In phase II trial IMMUTACE (NCT03572582), the procedure is associated with nivolumab administration in patients affected by intermediate-stage hepatocellular carcinoma. Moreover, in the phase II study TRIPLET (NCT04191889), the association of TACE with apatinib plus camrelizumab is under investigation in patients with C staged HCC, in BCLC classification. Even the phase II trial LEAP-012 (NCT04246177) is evaluating TACE combined with the administration of lenvatinib and pembrolizumab. In addition to the classic TACE (c-TACE), a variant is the drug-eluting bead transarterial chemoembolization (DEB-TACE). This type of procedure is also under investigation in combination with ICI, such as durvalumab and tremelimumab (NCT03638141) or nivolumab (NCT03143270).

A recent phase II study, NCT03259867 (TATE-PD1), involves the use of trans-arterial tirapazamine embolization (TATE) in patients with advanced HCC or other malignancies, simultaneously treated with nivolumab or pembrolizumab. The results of this new procedure are particularly interesting.

Considering radioembolization with yttrium 90 (Y90-RE), the results of a phase II, non-randomized trial (NCT03033446), and analyzing the combination with nivolumab in Asian advanced HCC patients, were recently presented. It enrolled 40 patients with a median follow-up of 16.4 months, and 36 patients were assessed. The combination of nivolumab plus Y90-RE resulted in an encouraging ORR of 31% (95%CI 16,4–48,1%), median PFS of 4.6 months (95%CI 2.3–4.8 months), and mOS of 15.1 months (95%CI 7.8–NE) (69). Furthermore, other trials are currently investigating Y90-RE in combination with nivolumab (NCT02837029) or pembrolizumab (NCT03099564).

In addition to the trials involving a single loco-regional procedure, several combination trials compare different methods. Among these, there is the phase III study NCT03949231 that confronts the hepatic artery infusion with the vein infusion of toripalimab (monoclonal anti-PD1 Ab) in patients with (BCLC) C-stage hepatocellular carcinoma. Furthermore, the phase II study NCT02821754 is estimating differences between chemoembolization (TACE), radiofrequency ablation (RFA), and cryoablation (CA) in patients with HCC and biliary tract cancer treated with tremelimumab and durvalumab. Another comparison study is the phase II trial NCT03753659, in which patients with early HCC received pembrolizumab and then underwent RFA versus Microwave Ablation (MWA).

## Immune Checkpoint Inhibitors + Tyrosine-Kinase Inhibitors (TKI)

HCC has a less dense vasculature with abnormal leaky and fragile tumor vessels, which lead to interstitial hypertension, tumor hypoxia, and necrosis (70–74). Hypoxia can, in turn, stimulate the angiogenic process, the tumor growth (71, 73, 75, 76), and may recruit immunosuppressive cells (77). Indeed, there is a complex bidirectional relationship between angiogenesis and immunity (78–88).

In particular, vascular endothelial growth factor (VEGF), in association with other pro-angiogenic determinants in the tumor microenvironment (TME), may down-regulate intercellular adhesion molecule 1 (ICAM-1) or vascular cell adhesion protein 1 (VCAM-1), repress T cell trafficking and dendritic cell (DC) maturation (77, 89). Moreover, the VEGF-A and pro-inflammatory cytokines cause Fas ligand (FasL) expression by tumor endothelial cells that gain the capacity to put CD8+ T cells but not T-reg cells to death (90). VEGF also increases PD-1 expression of tumor-infiltrating CD8+ T-cells (79). Also, PD-L1 expression is strongly dependent on transcriptional regulation of hypoxia-inducible factor 1-alpha (79, 91). Therefore, the blockade of the angiogenesis pathway might modify the immune TME, up-regulating CD8+ T-cells, and down-regulating immunosuppressor cells. That way, ICIs may improve the effectiveness of anti-angiogenic drugs inducing antibody-related cytotoxicity on endothelial cells. As a result, the destruction of the malignancy’s vasculature was obtained (92).

A Phase 1b study evaluated the safety and effectiveness of the association of durvalumab with ramucirumab, an anti-VEGF receptor-2 (VEGFR-2) IgG1 mAb, in different cohorts of advanced pre-treated cancer patients, including one cohort of 28 HCC subjects (NCT02572687). In the HCC cohort, ORR was 11%, but in patients that had “high” PD-L1 expression (≥25% of tumor cells or immune cells) achieved 18%. No significant differences in median PFS were observed accordingly to PD-L1 expression (4.4 in overall patients and 5.6 months in patients with high PD-L1 expression) as well as in mOS (10.7 and 16.5 months, respectively). Hypertension (17.9%), anemia (21.4%), and fatigue (10.7%) were the most frequent 3/4 TRAEs reported. Grade 3/4 TRAEs of interest reported in >5% of patients were hypertension, bleeding events (10.7%), and venous thromboembolic events (7.1%) for ramucirumab and lipase (10.6%) and AST increase (17.9%). Globally, the combination of durvalumab and ramucirumab did not show new safety signals and suggested potential anti-tumor activity, especially in the case of high PD-L1 expression. Further results are expected (93).

A multicenter, open-label, phase I/II dose-escalation and expansion study is assessing the harmlessness and benefit of MGD013, an anti-PD-1/anti-LAG-3 Dual-Affinity Re-Targeting (DART) protein in monotherapy and in combination with brivanib, a selective dual inhibitor of VEGFR and fibroblast growth factor receptors (FGFR) in advanced liver cancer patients (phase I- dose escalation also included intrahepatic cholangiocarcinoma) (NCT04212221).

Most TKIs have a remarkable anti-angiogenic effect through the inhibition of the VEGFRs (70) and have an immune-modulatory role as immune effectors involved in the TME and antigen presentation process (82). The association with ICI opens to the exploration of new treatment combinations to improve the anti-tumor immune response (94, 95).

Sorafenib is a multi-target TKI, approved since 2007 for first-line treatment of HCC, which can block the RAS, VEGFR, platelet-derived growth factor receptor (PDGFR), fms related tyrosine kinase 3 (FLT3), and KIT kinases, inducing apoptosis and blocking cell proliferation, migration, and cancer angiogenesis (96). Among the explored mechanisms of resistance to sorafenib in HCC, Liu et al. reported PD-L1 and DNA methyltransferases contribution (97). Currently, TKI and anti- PD-1 mAbs combination therapies were under study as first-line treatment for advanced HCC. In particular, the association with nivolumab is being assessed in a phase II, multicenter pilot trial in advanced HCC patients not eligible for surgery (NCT03439891). This trial will estimate the maximum tolerated dose, the safety, and ORR of the combination of sorafenib and nivolumab, along with the DOR, PFS, OS, peripheral and tumor immune cell profiling, PD-L1 expression, and alpha-fetoprotein (AFP) response (98).

A phase Ib/II study is evaluating sorafenib and pembrolizumab combination therapy in advanced HCC (NCT03211416). The primary endpoint is RR; secondary endpoints are safety, OS, and PFS. Moreover, the study will compare in blood and cancer samples the pre-treatment quantity of immunosuppressive cells and the functional activity of effector T cells post-treatment (99). Another phase Ib of dose-escalation and dose-expansion study is assessing the safety and tolerability of the combination of sorafenib with spartalizumab, an anti-PD-1 mAb, in advanced HCC (NCT02988440).

Lenvatinib is a small multi-TKI which works against VEGFR-1,-2, and -3, FGFR-1,-2,-3, and -4, PDGFRα, KIT, and (RET), approved on August 2018 by FDA for first-line treatment of unresectable HCC (100). Some ongoing clinical trials are studying its association with ICI.

The association between sorafenib and nivolumab is under evaluation in advanced HCC patients in two trials. In particular, a Japanese phase Ib trial aims to assess the tolerability and safety of this combination. Its secondary endpoints include OS, PFS, ORR, DOR, DCR, TTP, clinical Benefit Rate (CBR), and pharmacokinetics (PK) (NCT03418922). On the other hand, an exploratory, open-label, single-arm, multicenter phase II study evaluates the effectiveness and feasibility (as determined by safety and tolerability) of first-line sorafenib combined with nivolumab in patients with multinodular, advanced stage HCC. Primary endpoints are ORR, safety, and tolerability; secondary endpoints are TTP, PFS, OS, and translational research that consists of correlation of biomarkers potentially associated with clinical efficacy (NCT03841201-IMMUNIB).

Regarding the association of lenvatinib with pembrolizumab, preliminary data from a phase Ib study analyzing this combination in first-line setting for advanced HCC (NCT03006926) reported an ORR of 42.3%, and a median PFS of 9.69 months (95% CI 5.55–not evaluable). The most frequent any-grade TRAEs were decreased appetite and hypertension (53.3% each), diarrhea (43.3%), and fatigue (40%). The most common grade ≥3 TRAEs described were hypertension (16.7%), aspartate aminotransferase (AST) increment (16.7%), neutropenia (13.3%), and hyponatremia (10.0%). Eight patients had severe adverse events (SAEs) (26.7%), and 16.7% discontinued lenvatinib and or pembrolizumab due to TRAEs, but side effects were controlled (101).

Based on these results, the phase III multicenter, randomized, double-blinded, active-controlled, LEAP-002 trial (NCT03713593) is testing the effectiveness and safety of lenvatinib and pembrolizumab combination therapy versus lenvatinib combined with placebo as first-line treatment in advanced HCC Child-Pugh class A patients. This trial estimates to randomize 750 patients approximately. The primary endpoints are OS and PFS, whereas secondary endpoints include ORR, DOR, DCR, TTP, adverse events, and PK (102). Also, a single-arm phase IIb study is assessing lenvatinib and pembrolizumab combination therapy as second-line treatment in patients with unresectable hepatobiliary tumors, including the analysis of potential biomarkers of response (NCT03895970).

Regorafenib is a multi-target TKI that actively suppresses VEGFR-1,-2,-3, PDGFR, TIE-2, fibroblast growth factor receptor 1 (FGFR1), KIT (CD117), RET, and B-Raf (103). It is under evaluation in combination with ICI in two ongoing studies.

A multicenter, non-randomized, open-label, dose-escalation, phase Ib study is assessing the harmlessness and tolerability of the association of regorafenib and pembrolizumab as first-line treatment for patients with advanced HCC (NCT03347292). Moreover, the study aims to explore the anti-tumor activity of this combination and to determine blood/tissue biomarkers related to the tumor activity, status or response.

The REGOMUNE trial (NCT03475953) is a multicenter phase I/II trial which is estimating the combination of regorafenib and avelumab in solid tumors, including HCC, after at least one previous line of systemic therapy. Phase I will establish the recommended phase II dose (RP2D), whereas phase II will assess the efficacy and safety of the drugs combination.

Cabozantinib is a TKI targeting VEGFR-2, c-MET, AXL, RET and FLT-3 (100, 104). One cohort of the Checkmate040 phase I/II trial (NCT01658878) is assessing the potential synergistic activity of cabozantinib combined with nivolumab, with or without ipilimumab, in Child-Pugh A advanced HCC patients; primary endpoints are safety and ORR (29, 105, 106).

A phase Ib, open-label trial will explore the safety, tolerability, preliminary efficacy, and PK of cabozantinib combined with atezolizumab in advanced HCC patients (NCT03170960). In the dose-escalation phase (3 + 3 design), a recommended dose for cabozantinib and atezolizumab combination therapy will be determined. In the expansion phase, 18 cohorts will be recruited at the recommended dose of cabozantinib and atezolizumab, comprising one cohort of advanced systemic-treatment naïve HCC. The primary objective is the ORR for each cohort (107).

The phase III COSMIC-312 trial (NCT03755791) is appraising cabozantinib plus atezolizumab versus sorafenib in the first-line setting in advanced HCC patients, Child-Pugh A. Patients will be randomized in a 2:1:1 ratio to take cabozantinib plus atezolizumab, sorafenib, or single-agent cabozantinib. The study has two primary endpoints: compare OS and PFS for cabozantinib + atezolizumab versus sorafenib; the secondary endpoint is PFS for cabozantinib versus sorafenib (108).

The open-label, single-arm, CAMILLA trial is a phase Ib study of cabozantinib and durvalumab combination therapy in pretreated patients with advanced HCC (NCT03539822). The study intends to examine the safety and tolerability and display preliminary data on effectiveness (109).

Axitinib is a TKI selective for VEGFR-1/2/3. VEGF Liver 100 (NCT03289533) is a Phase Ib study assessing the feasibility of the combination of avelumab plus axitinib in treatment-naive patients with HCC in terms of harmlessness and effectiveness. Provisory results of the analysis showed an ORR of 13.6% based on RECIST 1.1 and 31.8% based on mRECIST criteria. mPFS was 5.5 and 3.8 months, according to RECIST and mRECIST, respectively. Tumor shrinkage was reported in 68.2% of patients by RECIST and 72.7% of patients by mRECIST. OS data were still immature. The most common grade 3 TRAEs were hypertension (50.0%) and hand-foot syndrome (22.7%); no grade 4/5 TRAEs were mentioned. Immune-related AEs (irAEs) occurring in ≥10% of patients were hypothyroidism (31.8%) and hyperthyroidism (13.6%). None of irAEs were grade ≥3. No treatment discontinuations due to TRAEs or irAEs were registered. Thus, safety and efficacy results were promising, but further follow-up is required (110).

Apatinib is an impressive TKI inhibitor of VEGFR-2, c-Kit, c-Src, and PDGFR. An open-label, dose-escalation (phase Ia) and expansion study (phase Ib) evaluated the safety and efficacy of the camrelizumab, an anti-PD-1 mAb, and apatinib combination therapy in advanced HCC patients (NCT02942329). The main goals were harmlessness and tolerability and RP2D determination. A grade 3 TRAE was reported in 60.6%. Hypertension (15.2%) and elevated AST (15.2%) were the most common. Results showed that camrelizumab and apatinib combination had a feasible safety profile and activity against cancer cells in HCC patients (111). The phase II, single-arm, RESCUE study (NCT03463876) is preliminary exploring the efficacy and safety of the combination of apatinib and camrelizumab regimen as second-line treatment in advanced HCC; the primary endpoint is ORR.

Currently, is ongoing a randomized, open-label, international, multicenter, phase III trial of camrelizumab plus apatinib versus sorafenib in first-line setting in patients with unresectable HCC that did not receive systemic treatment in the past (NCT03764293). The co-primary endpoints are OS and PFS.

## Immune Checkpoint Inhibitors + C-Met Inhibitors

The MET/HGF pathway stimulate cellular proliferation, survival, and invasion and progression in HCC and has been associated with TKI resistance (112–114). A phase Ib/II, open-label, multicenter study is assessing the association of capmatinib (INC280), a selective oral c-MET recently developed in HCC, and spartalizumab versus spartalizumab single-agent in advanced HCC patients, progressing after sorafenib (NCT02795429).

Another phase I/II dose-escalation, and expansion study is testing bozitinib, a c-MET inhibitor, combined with genolimzumab, an anti-PD-1 mAb, after first-line treatment for locally advanced or unresectable HCC not pretreated with a PD-1 inhibitor or a c-MET inhibitor (NCT03655613).

## Immune Checkpoint Inhibitors + FGFR Inhibitors

Another promising approach is represented by the association of ICI with inhibitors of the fibroblast growth factor 19 (FGF19)/FGF receptor 4 (FGFR4) pathway (115). The alteration of the FGF19/FGFR4 signaling is a known driver of HCC carcinogenesis (116). It suppresses E-cadherin expression and promotes the expression of epithelial-to-mesenchymal transition (EMT)-related genes, leading to increased HCC cell invasion. FGF19/FGFR4 axis has been associated with poor prognosis. Moreover, FGF19 expression has been related with early relapse and shorter disease-specific recurrence in a cohort of resected HCC patients and appears implicated in sorafenib resistance (117, 118).

A Phase I/II, multicenter, open-label study is assessing the combination of oral FGF401, an FGFR4 inhibitor, with spartalizumab in refractory HCC patients harboring FGFR4 and KLB (an FGF19 co- receptor) expression and FGF401 as single-agent in other advanced solid tumors. The study is investigating the efficacy as the dose-limiting toxicity to detect the maximum tolerated dose and/or RP2D (NCT02325739).

## Immune Checkpoint Inhibitors + TGFβ Pathway Inhibitors

TGF-β contributes to cell invasion, angiogenesis, EMT, and drug resistance in HCC, as demonstrated by several preclinical findings (119–121). Moreover, TGF-β may induce *in vitro* FGFR4 expression through the extracellular-signal-regulated kinase (ERK) pathway, and its interaction with FGFR4 promotes the metastatic spread of HCC *in vivo* (122). TGF-β also plays a critical role in HCC immune-tolerance. Indeed, it is secreted by Kupffer cells and liver sinusoidal endothelial cells, and it can up-regulate the Treg, and recently, Mariathasan et al. reported that TGF-β weakened tumor response to PD-L1 inhibition by contributing to exclude T cells (123–127). For these reasons, a combined approach of the TGF-β pathway and PD-1/PD-L1 inhibitors, or a managing bifunctional fusion proteins targeting both TGF-β and PD-L1, might overcome drug resistance and have a synergistic effect (128–130).

Galunisertib (LY2157299 Monohydrate) is an oral TGF-β receptor-1 (TGF-βR1) inhibitor that showed a favorable safety profile as single-agent or in combination with sorafenib (131). Currently, galunisertib is under investigation in combination with nivolumab in a phase Ib/II (dose escalation and cohort expansion) study in advanced solid tumors, including HCC with AFP ≥200 ng/ml, as second-line treatment. The main goal of this study is to estimate the harmlessness, tolerability, and effectiveness of this drug association (NCT02423343).

A phase I/Ib, open-label, multi-center, dose-escalation ongoing trial is assessing the safety and tolerability of NIS793, a novel anti-TGF-β antibody (Ab) alone or in combination with spartalizumab in advanced refractory solid tumors, including HCC (NCT02947165). The study also aims to identify recommended doses and schedules of these drugs (NIS793: every 2 or every 3 weeks; spartalizumab: every 3 or 4 weeks) for future studies.

Another promising approach for the future might be M7824 (MSB0011359C), an innovative first- in-class bifunctional fusion protein that consists of a human IgG1 anti-PD-L1 mAb (avelumab) fused to the extracellular domain of TGFβ receptor II (TGF-βRII) to act as a TGFβ “trap”. Results of a phase I dose-escalation study with M7824 showed an amenable safety profile in heavily pre- treated patients with advanced solid tumors. Multiple expansion cohorts are ongoing in various tumor types (NCT02517398) (132).

## Immune Checkpoint Inhibitors + Chemotherapy

The EACH trial, a randomized, multicenter, open-label study of palliative FOLFOX versus doxorubicin in Asian patients with advanced HCC, has led the China FDA to introduce FOLFOX4 in the clinical practice guideline (PR 8.6%, 38.6% SD, median OS 5.7 months) (133).

It has been reported that oxaliplatin can induce an anti-tumor immune response and immunogenic cell death, more specifically by activation of DCs, the enhancement of cross-priming of CD8- positive (CD8+) T cells, the stimulation of the anti-tumor CD4+ T cells phenotype, and down- regulation of MDSC and T-reg cells. Moreover, oxaliplatin promotes tumor cell death through lytic receptors/pathways, boosted serum inflammatory cytokines, and switch to pro-inflammatory status in the TME (133, 134). A Phase II, non-randomized study is assessing the combination of camrelizumab with apatinib or with chemotherapy in patients with advanced HCC (FOLFOX4) who failed or were unbearable to prior systemic therapy (NCT03092895).

## Future Perspectives

It is well-known that in some patients, due to the lack of tumor-infiltrating effector T cells, checkpoint inhibitors were ineffective. However, cancer vaccines seem to be able to increase effector T-cells infiltration into tumors. Therefore, a strategy combining a cancer vaccine with an immune checkpoint inhibitor may be promising. The synergistic action of the two drugs may lead to an effective antitumor immune response: whilst the vaccine raises the number of tumor-infiltrating effector T cells, the anti-PD-1 makes sure that these cells stay active (135). Hence, clinical trials are warranted.

## Discussion

In the last few years, several studies evaluated new drug combinations (134, 136). These new therapeutic approaches could soon make a difference.

As for the adjuvant setting, there are no available data up to now, but there are several phase III trials ongoing on various immunocheckpoint inhibitors. We will look forward to the results of these studies, which would seem to prospect the best disease control rate. If data will be statistically significant, we will make a relevant step forward. Anyhow, for now, in the localized HCC, surgery represents the standard of care (**Table 1**).

**Table 1:** Adjuvant ICI: ongoing and still recruiting clinical trials.

Drug	Trial name	Phase	Design	Endpoint	N	Start date	ClinicalTrials.gov	Status
Toripalimab	JUPITER 04	II/III	Toripalimab vs placebo	RFS	402	01/03/2019	NCT03859128	Recruiting
Nivolumab	CheckMate 9DX	III	Nivolumab vs placebo	RFS	530	18/12/2017	NCT03383458	Recruiting
Durvalumab	EMERALD-2	III	Durvalumab + bevacizumab (arm A); Durvalumab + placebo (arm B); placebo + placebo (arm C);	RFS (arm B vs arm C)	888	29/04/2019	NCT03847428	Recruiting
Pembrolizumab	KEYNOTE-937	III	Pembrolizumab vs placebo	RFS and OS	950	28/05/2019	NCT03867084	Recruiting
Atezolizumab	IMbrave050	III	Atezolizumab + bevacizumab (arm A); active surveillance (arm B);	RFS	662	31/12/2019	NCT04102098	Recruiting

Regarding the combination of locoregional treatments and immunocheckpoint inhibitors, several phase II trials are underway. There is only a phase III trial on Toriliplimab, but no data is available yet. The unique existing data are related to a small cohort. Thus, the results are not reliable (**Table 2**).

**Table 2:** Ongoing trials on loco-regional treatments of unresectable HCC.

Phase	Drugs	Procedure	Setting	NCT

**III**	Toripalimab	Hepatic artery versus vein infusion of Toripalimab.	(BCLC)-C-stage Hepatocellular Carcinoma (HCC)	NCT03949231
**II/III**	Sintilimab	Stereotactic body radiotherapy (SBRT)	Advanced hepatocellular carcinoma (HCC)	NCT04167293(ISBRT01)
**II/III**	Pembrolizumab and/or ipilimumab	Trans-artery/intra-tumor infusion	Solid tumors (including hepatocellular carcinoma)	NCT03755739
**II**	Nivolumab	Transarterial Chemoembolization (TACE)	Intermediate Stage Hepatocellular Carcinoma	NCT03572582(IMMUTACE)
**II**	tremelimumab and durvalumab	Chemoembolization (TACE), radiofrequency ablation (RFA) and cryoablation (CA)	Advanced hepatocellular carcinoma (HCC) and biliary tract carcinomas (BTC)	NCT02821754
**II**	Nivolumab	Y90-Radioembolization	Asians with hepatocellular carcinoma	NCT03033446
**II**	Nivolumab	Selective internal radiation therapy (SIRT)	Advanced hepatocellular carcinoma (HCC)	NCT03380130(NASIR-HCC)
**II**	Apatinib and Camrelizumab	Chemoembolization (TACE)	C staged Hepatocellular Carcinoma in BCLC classification	NCT04191889(TRIPLET)
**II**	Pembrolizumab	Radio frequency ablation (RFA), microwave ablation (MWA)	Early stage hepatocellular carcinoma (HCC)	NCT03753659
**II**	nivolumab or pembrolizumab	Trans-arterial Tirapazamine Embolization (TATE)	Hepatocellular carcinoma (HCC), metastatic colorectal cancer (mCRC), metastatic gastric cancer and advanced non-small cell lung cancer	NCT03259867(TATE-PD1)
**II**	Durvalumab and Tremelimumab	Drug-eluting bead transarterial chemoembolization (DEB-TACE)	Newly diagnosed with hepatocellular carcinoma	NCT03638141
**II**	JS001 (Terepril) and Apatinib	Stereotactic body radiotherapy (SBRT)	BCLC stage C hepatocellular carcinoma (HCC) with PVTT	NCT04165174
**II**	PD-1 mAb and lenvatinib	Chemoembolization (TACE)	Middle and late stage (BCLC-B and BCLC-C) HCC patients	NCT04273100
**II**	Carrizumab and Apatinib	Radiofrequency ablation (RFA)	Advanced hepatocellular carcinoma (HCC)	NCT04150744
**II**	Lenvatinib and Pembrolizumab	Transarterial Chemoembolization (TACE)	Advanced hepatocellular carcinoma (HCC)	NCT04246177(LEAP-012)
**II**	PD-1 mAb	TACE, SBRT	Neoadjuvant HCC	NCT03817736
**II**	Anti-PD-1 Antibody (IBI308)	Stereotactic body radiation therapy (SBRT)	Advanced hepatocellular carcinoma (HCC)	NCT03857815
**II**	Pembrolizumab	Stereotactic body radiotherapy (SBRT)	Advanced hepatocellular carcinoma (HCC)	NCT03316872
**II**	Sintilimab	Transarterial chemoembolization (TACE)	Advanced hepatocellular carcinoma (HCC) as first-line therapy	NCT04297280
**II**	Sintilimab and FOLFOX	Hepatic arterial infusion chemotherapy (TAI)	Locally advanced, potentially resectable HCC	NCT03869034
**I/II**	Toriplimab	Radiofrequency ablation (RFA)/microwave ablation (MWA)	Advanced hepatocellular carcinoma (HCC)	NCT03864211
**I**	Nivolumab	Drug eluting bead transarterial chemoembolization (deb-TACE)	Advanced hepatocellular carcinoma (HCC)	NCT03143270
**I**	Sintilimab	Microwave ablation, TACE	Advanced hepatocellular carcinoma (HCC)	NCT04220944
**I**	ImiquimodDrug: Standard of Care PD-1 Therapy	Focused ultrasound ablation (FUSA)	Solid tumors (including hepatocellular carcinoma)	NCT04116320(AM-003)
**I**	Nivolumab	Yttrium Y 90 glass microspheres	Stage III-IV hepatocellular carcinoma (HCC)	NCT02837029
**I**	Pembrolizumab	Y90 radioembolization	Hepatocellular carcinoma (HCC)	NCT03099564
**I**	Sintilimab	Radiotherapy	HCC with main portal vein tumor thrombosis	NCT04104074

Nonetheless, the available evidence suggests that combining systemic therapies and locoregional treatments with immune checkpoint inhibitors may represent a useful strategy in this context.

In the advanced HCC, thanks to the improvement of OS, PFS, and QoL achieved by the phase III IMbrave150 trial, the FDA approved atezolizumab + bevacizumab as first-line therapy in this setting (26).

Another drug that seems to be promising is tremelimumab, but we are looking forward to the phase III Himalaya trial results. This trial is assessing the combination of tremelimumab + durvalumab.

As for anti-PD-1, nivolumab and pembrolizumab, there are controversial results. Based on the results of phase II trials (CheckMate 040 and Keynote 224), the FDA approved nivolumab and pembrolizumab for advanced HCC. However, the phase III trials (CheckMate 459 and Keynote 240) did not match up to their primary endpoints of OS and PFS. Nonetheless, there are some aspects to take into consideration. CheckMate 040 was a non-comparative study on advanced HCC patients not all pre-treated with Sorafenib. On the other hand, CheckMate 459 compared Nivolumab with Sorafenib in the first-line setting. Although the design of the studies was different, phase III data were interesting thanks to the best tolerability of the drug in the patients, along with a positive trend in terms of response rate and overall survival. Likewise, the Keynote 224 examined the use of Pembrolizumab in 104 advanced HCC patients pre-treated with Sorafenib, whereas the Keynote 240 analyzed pembrolizumab vs placebo in 413 patients as second line treatment. Maybe a first-line setting could have different outcomes or maybe an enlarged sample of patients might have led to different results. Even so, the patients did not suffer the side effects as well as an improvement in survival and response rate. Therefore, taking in consideration the QoL of the patients the approval of these drugs was considerate.

Also, due to the promising results of the combination of nivolumab + ipilimumab, analyzed in cohort 4 in phase II CheckMate 040 trial, the FDA approved them for usage in clinical practice.

No phase III trials are ongoing, so they are warranted (**Tables 3** and **4**).

**Table 3:** Clinical Trials in Advanced HCC.

.Drug	Trial name	Phase	Design	Endpoint	N	Start date	ClinicalTrials.gov	Status
Tremelimumab		II	Tremelimumab	ORR	20	December 2008	NCT01008358	Completed
Durvalumab, tremelimumab		II	Tremelimumab; Durvalumab; Tremelimumab 300 + Durvalumab; Tremelimumab 75 + Durvalumab	Safety, tolerability, and activity	433	19/10/2015	NCT02519348	Active, not recruiting
Durvalumab, tremelimumab	Himalaya	III	Durvalumab vs tremelimumab + durvalumab vs sorafenib	OS	1324	11/10/2017	NCT03298451	Active, not recruiting
Nivolumab, ipilimumab, cabozantinib	CheckMate 040	I/II	Nivolumab; nivolumab + ipilumumab; nivolumab + cabozantinib; nivolumab + ipilimumab + cabozantinib; sorafenib	ORR	1097	26/09/2012	NCT01658878	Active, not recruiting
Nivolumab	CheckMate 459	III	Nivolumab vs sorafenib	OS	743	25/11/2015	NCT02576509	Active, not recruiting
Pembrolizumab	KEYNOTE-224	II	Pembrolizumab	ORR	104	31/05/2016	NCT02702414	Active, not recruiting
Pembrolizumab	KEYNOTE-240	III	Pembrolizumab vs placebo	PFS and OS	413	26/05/2016	NCT02702401	Active, not recruiting
Atezolizumab, bevacizumab	GO30140	Ib	Atezolizumab + bevacizumab; atezolizumab	ORR and PFS	223	06/04/2016	NCT02715531	Active, not recruiting
Atezolizumab	IMbrave150	III	Atezolizumab + bevacizumab vs sorafenib	OS and PFS	501	15/03/2018	NCT03434379	Active, not recruiting

**Table 4:** ICI + Target Therapies Clinical trials for Advanced HCC patients.

Phase	Drugs	Molecular Target	Setting	NCT

**Ib**	Durvalumab + Ramucirumab	Tyrosine-kinase inhibitor	Advanced pre-treated HCC	NCT02572687
**I/II**	MGD013; MGD013 + brivanib	Tyrosine-kinase inhibitor	Advanced liver cancer patients	NCT04212221
**II**	Sorafenib + Nivolumab	Tyrosine-kinase inhibitor	1^st^ line in Advanced HCC	NCT03439891
**Ib/II**	Sorafenib + Pembrolizumab	Tyrosine-kinase inhibitor	Advanced HCC	NCT03211416
**Ib**	Sorafenib + Spartalizumab	Tyrosine-kinase inhibitor	Advanced HCC	NCT02988440
**Ib**	Sorafenib + Nivolumab	Tyrosine-kinase inhibitor	Advanced HCC	NCT03418922
**II**	Sorafenib + Nivolumab	Tyrosine-kinase inhibitor	Advanced HCC	NCT03841201-IMMUNIB
**Ib**	Lenvatinib + Pembrolizumab	Tyrosine-kinase inhibitor	Advanced HCC	NCT03006926
**III**	Lenvatinib + Pembrolizumab vs Lenvatinib + placebo	Tyrosine-kinase inhibitor	1^st^ line in Advanced HCC	NCT03713593 – LEAP-002
**IIb**	Lenvatinib + Pembrolizumab	Tyrosine-kinase inhibitor	2^nd^ line unresectable Hepatobiliary cancers	NCT03895970
**Ib**	Regorafenib + Pembrolizumab	Tyrosine-kinase inhibitor	1^st^ line in Advanced HCC	NCT03347292
**I/II**	Regorafenib + Avelumab	Tyrosine-kinase inhibitor	2^nd^ line Advanced HCC	NCT03475953 -REGOMUNE
**I/II**	Cabozantinib + Nivolumab	Tyrosine-kinase inhibitor	Advanced HCC	NCT01658878 – CheckMate 040
**Ib**	Cabozantinib + Atezolizumab	Tyrosine-kinase inhibitor	Advanced HCC	NCT03170960
**III**	Cabozantinib + Atezolizumab	Tyrosine-kinase inhibitor	1^st^ line in Advanced HCC	NCT03755791 - COSMIC-312
**Ib**	Cabozantinib + Durvalumab	Tyrosine-kinase inhibitor	Pretreated Advanced Cancer	NCT03539822 - CAMILLA
**Ib**	Axitinib + Avelumab	Tyrosine-kinase inhibitor	Treatment-naive HCC patients	NCT03289533 – VEGF Liver 100
**Ia/Ib**	Apatinib + Camrelizumab	Tyrosine-kinase inhibitor	Advanced HCC	NCT02942329
**II**	Apatinib + Camrelizumab	Tyrosine-kinase inhibitor	2^nd^ line Advanced HCC	NCT03463876 - RESCUE
**III**	Apatinib + Camrelizumab vs Sorafenib	Tyrosine-kinase inhibitor	1^st^ line in unresectable Advanced HCC	NCT03764293
**Ib/II**	Capmatinib + Spartalizumab vs Spartalizumab	c-MET inhibitor	2^nd^ line Advanced HCC after progression to Sorafenib	NCT02795429
**I/II**	Bozitinib + Genolimzumab	C-MET inhibitor	2^nd^ line for locally advanced or unresectable HCC	NCT03655613
**I/II**	FGF401 + Spartalizumab	FGFR inhibitor	in refractory HCC patients harboring FGFR4 and KLB	NCT02325739
**Ib/II**	Galunisertib + Nivolumab	TGF-βR1 inhibitor	Advanced HCC	NCT02423343
**I/Ib**	NIS793 vs NIS793 + Spartalizumab	Anti-TGF-β Antibody	Advanced refractory HCC	NCT02947165
**I**	M7824	A TGFβ "trap"	Heavily pre-treated patients with Advanced Cancer	NCT02517398
**II**	Apatinib + Camrelizumab vs Chemotherapy + Camrelizumab	Tyrosine-kinase inhibitor	Advanced Cancer	NCT03092895

Many studies are analyzing the combination of ICI + TKI in the first-line in the metastatic setting. A few of them are phase III trials such as the LEAP-002 trial that is evaluating lenvatinib + pembrolizumab versus placebo, whereas the COSMIC-312 trial is assessing cabozantinib + atezolizumab versus sorafenib as the NCT03764293 trial camrelizumab + apatinib versus sorafenib. Their results were awaited. Other combinations of ICI with target therapies as C-Met, FGFR, and TGF-β, are understudy for the second-line in advanced HCC. However, they are still phase I or II trials. For sure, these emerging combinations represent the most promising therapies so far, on which we could rely more in the future.

Also, a combination of chemotherapy, oxaliplatin, and ICI is evaluating in phase II trials based on the role that oxaliplatin plays in promoting the action of immunotherapy.

However, it appears clear that we should opt for combining therapies over a single-agent treatment to overcome the drug-resistance. Nevertheless, in order to tailor a therapy that fits the single patient perfectly, we need to determine some specified biomarkers.

In conclusion, given the encouraging results emerging from the preliminary data of some phase I-II trials, and waiting for the results of the ongoing studies, it is possible to hope that some agents can be successfully combined in the second-line as well as in the first-line. Indeed, these new promising therapeutic options may soon change the clinical practice. Nonetheless, other clinical trials are needed to define a better treatment sequence.

## Author Contributions

All authors listed have made a substantial, direct, and intellectual contribution to the work and approved it for publication.

## Conflict of Interest

The authors declare that the research was conducted in the absence of any commercial or financial relationships that could be construed as a potential conflict of interest.
